# Acquired Peripheral Nerve Injury Findings in Critically Ill COVID-19 Patients

**DOI:** 10.5334/jbsr.2757

**Published:** 2022-04-28

**Authors:** Louise Torres, Ângela Massignan, Ramon Gheno, Jônatas Fávero Prietto dos Santos, Marcelo Vianna Raffo, Guilherme Jaquet Ribeiro

**Affiliations:** 1 Musculoskeletal Imaging Fellowship Program, Hospital Moinhos de Vento, Porto Alegre, BR; 2 Musculoskeletal Radiologist, Hospital Moinhos de Vento, Porto Alegre, BR; 3 Radiology and Diagnostic Imaging Residency Program, Hospital Moinhos de Vento, Porto Alegre, BR; 4 Neurologist, Hospital Moinhos de Vento, Porto Alegre, BR

**Keywords:** Covid-19, peripheral neuropathy, electromyography, MR neurography, MRI

## Abstract

We retrospectively analyzed clinical, NCS/EMG, and NMRI aspects of five COVID-19 intensive care unit inpatients that received mechanical ventilation. After awakening from sedation, they experienced peripheral neuromyopathic symptoms.

**Teaching Point:** Acquired peripheral nerve injury has been described in COVID-19 infection and knowledge of the clinical, nerve conduction studies/electromyography (NCS/EMG) and neurographic magnetic resonance imaging (NMRI) findings are crucial.

## Introduction

Neurological complications have been reported in critically ill COVID-19 patients [1,2,3]. Clinical, nerve conduction studies/electromyography (NCS/EMG), and neurographic magnetic resonance imaging (NMRI) data from five patients with severe COVID-19 and peripheral neuropathy were retrospectively reviewed.

## Case History 1

**Figure 1:**
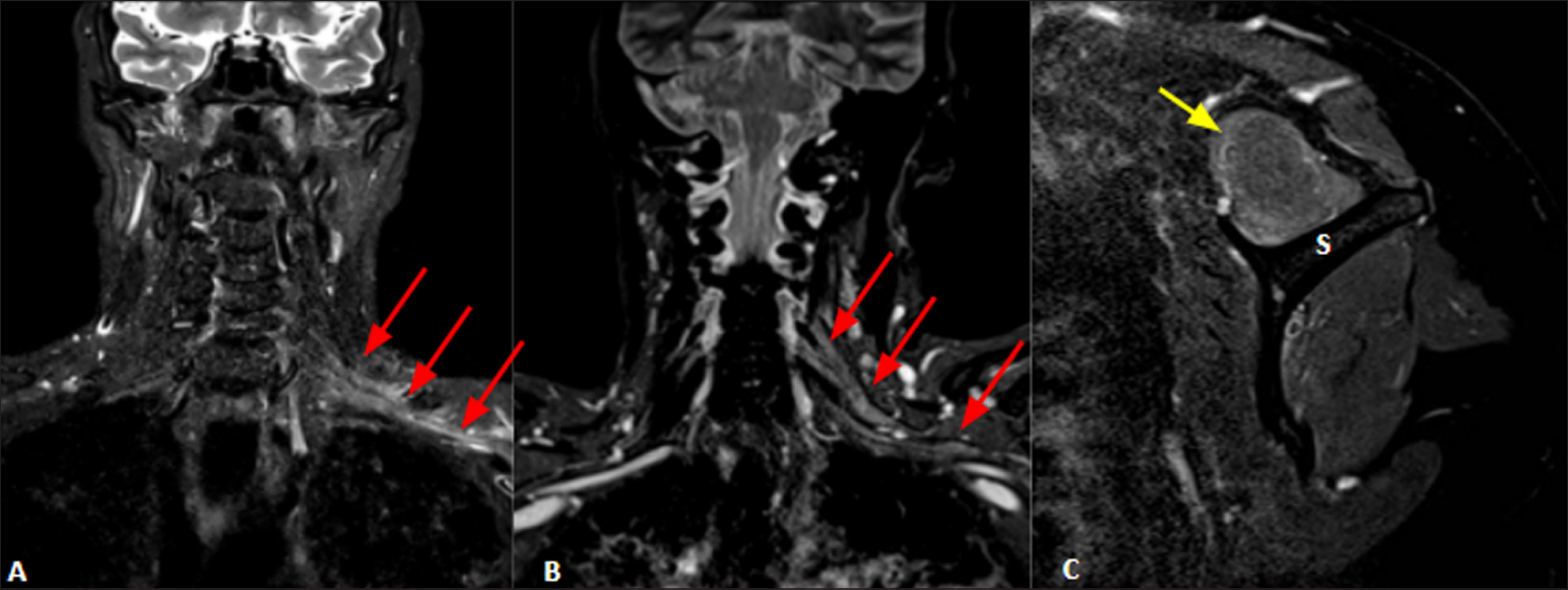
Coronal T2WI (**A** and **B**) of two different patients shows thickening and increased signal intensity in trunks, divisions, chords, and terminal branches of the left brachial plexus (red arrows), suggesting neuritis. Left shoulder sagittal T2WI (**C** – same patient in B), depicts high signal intensity in the supraspinatus muscle (yellow arrow), due to edema/denervation. S = scapular spine.

A 48-year-old woman presented with sudden onset of severe burning pain and progressive weakness in the left shoulder girdle. NCS/EMG suggested acute motor sensory axonal neuropathy (AMSAN) and left brachial neuritis. Left brachial plexus NMRI was consistent with Parsonage-Turner syndrome (***Figure 1B*** and ***1C***).

## Case History 2

A 69-year-old man presented with flaccid quadriplegia, upper limbs areflexia and lower limbs hyporeflexia. NCS/EMG suggested a sensorimotor polyneuropathy (attributed to critical illness polyneuropathy) and Parsonage-Turner syndrome, confirmed by NMRI.

## Case History 3

A 78-year-old woman presented with prone positioning for two days. Quadriplegia due to critical illness polyneuropathy and left shoulder pain was present after awakening from sedation. NCS/EMG and NMRI were consistent with Parsonage-Turner syndrome (***Figure 1A***).

## Case History 4

**Figure 2:**
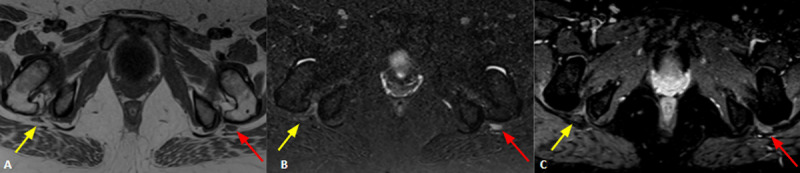
Axial T1WI **(A)**, T2WI with fat suppression **(B)** and post contrast T1WI **(C)** show asymmetry of the extra-pelvic sciatic nerves, depicting increased signal intensity in T2WI, thickening and enhancement of the left sciatic nerve (red arrows), suggesting neuritis. Note the normal right sciatic nerve (yellow arrows).

A 35-year-old woman presented with anesthesia and distal left leg muscle strength grade 0. Lumbosacral plexus MRI revealed signs of sciatic neuritis (***Figure 2***). Mononeuropathy related to COVID-19 was diagnosed.

## Case History 5

**Figure 3:**
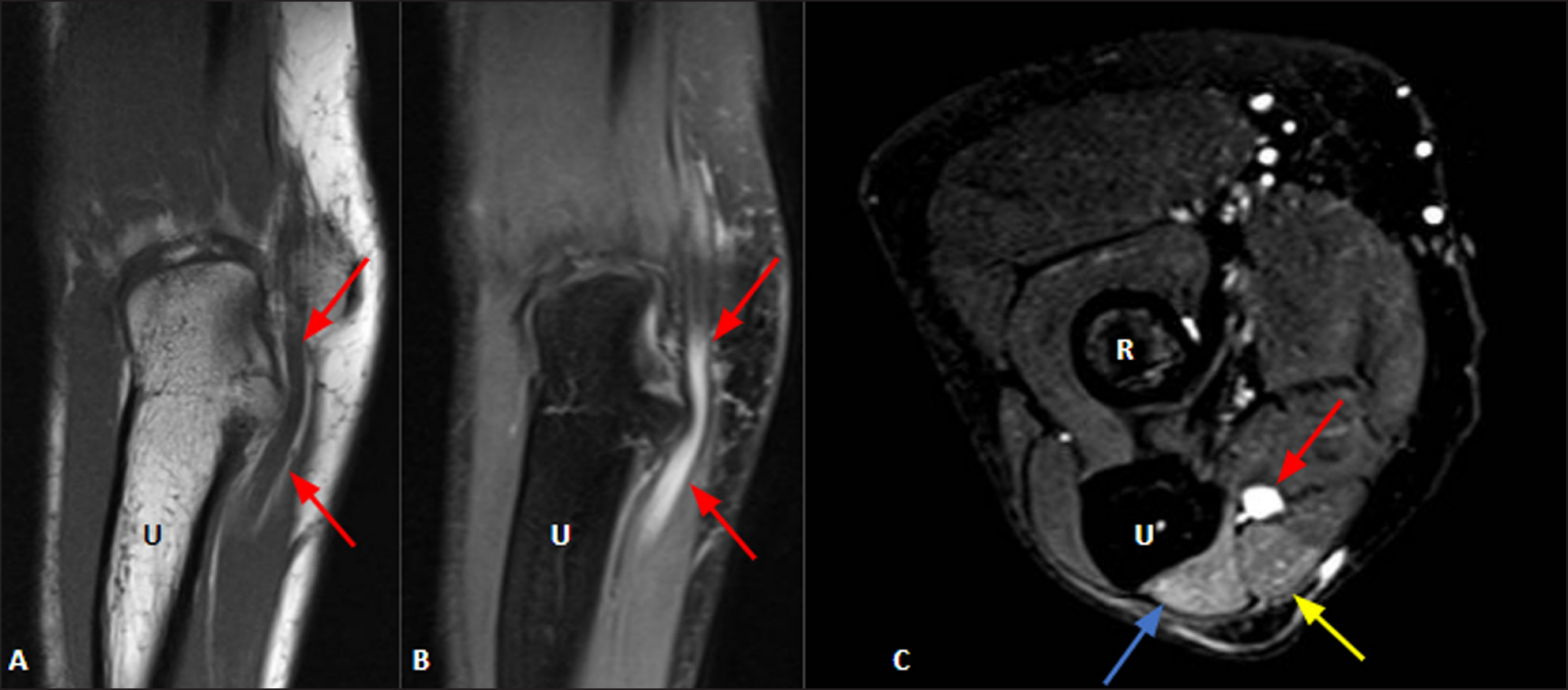
Coronal T1WI **(A)** and corresponding coronal T2WI **(B)** with fat suppression show ulnar nerve thickening and T2WI increased signal intensity at the level and distal to cubital tunnel (red arrows), suggesting ulnar neuropathy. Axial T2WI with fat suppression **(C)** depicts ulnar nerve thickening and increased signal intensity (red arrow), also as high signal intensity in the flexor carpi ulnaris (yellow arrow) and flexor digitorum profundus (blue arrow) due to edema/denervation. R = radius; U = ulna.

A 48-year-old woman presented with numbness along the fifth and fourth left fingers, weakness and volume loss between first and second metacarpal bones. Elbow MRI was consistent with ulnar neuropathy (***Figure 3***), attributed to ulnar nerve compression lesion.

## Comment

COVID-19’s related neuropathy is not yet completely understood. Nevertheless, the receptor for SARS-CoV-2 (angiotensin converting enzyme 2) has expression in the nervous system [1,4].

Post-infectious inflammatory peripheral nerve injury is thought to be immune-mediated and occurs in the setting of several viruses [1]. Peripheral nerve injury has been described following the use of prone positioning for COVID-19-related acute respiratory distress syndrome [1,5,6,7,8] and brachial plexus is at greater risk [5,9], as a result of traction during decubitus changes and stretch/compression injury from prolonged prone positioning [1,9]. Positioning-related peripheral nerve injury typically results in neuropraxia or axonotmesis [1,6]. Corresponding NMRI findings include nerve signal hyperintensity, thickening, and sometimes, fascicular enlargement. Polyneuropathy and critical illness myopathy are well-known complications of critical care treatment [10]. COVID-19 patients may have critical illness myopathy superimposed with peripheral nerve injury. MRI may show a pattern of muscle edema correlated with expected distribution of innervation of the affected peripheral nerve [1].

## Conclusions

Clinical, NCS/EMG, and NMRI are important to assess the potential etiologies and severity of peripheral nerve injury. Early diagnosis may guide treatment decisions, which could improve the clinical outcome.
